# Unsupervised Classification of English Words Based on Phonological Information: Discovery of Germanic and Latinate Clusters

**DOI:** 10.1162/OPMI.a.346

**Published:** 2026-04-07

**Authors:** Takashi Morita, Timothy J. O’Donnell

**Affiliations:** Academy of Emerging Sciences, Chubu University, Kasugai, Japan; Institute for Advanced Research, Nagoya University, Nagoya, Japan; Department of Linguistics, McGill University, Montréal, Canada; Canada CIFAR AI Chair, Mila, Montréal, Canada; Mila—Quebec AI Institute, Montréal, Canada

**Keywords:** phonotactics, English etymology, double object construction, Bayesian learning, learnability

## Abstract

Cross-linguistically, native words and loanwords follow different phonological rules. In English, for example, words of Germanic and Latinate origin exhibit different stress patterns, and a certain syntactic structure—double-object datives—is predominantly associated with Germanic verbs rather than Latinate verbs. From the perspective of language acquisition, however, such etymology-based generalizations raise learnability concerns, since the historical origins of words are presumably inaccessible information for general language learners. In this study, we present computational evidence indicating that the Germanic-Latinate distinction in the English lexicon is learnable from the phonotactic information of individual words. Specifically, we performed an unsupervised clustering on corpus-extracted words, and the resulting word clusters largely aligned with the etymological distinction. The model-discovered clusters also recovered various linguistic generalizations documented in the previous literature regarding the corresponding etymological classes. Moreover, our model also uncovered previously unrecognized features of the quasi-etymological clusters. Taken together with prior results from Japanese, our findings indicate that the proposed method provides a general, cross-linguistic approach to discovering etymological structure from phonotactic cues in the lexicon.

## INTRODUCTION

Discovering appropriate groups of observations without access to correct answers (i.e., unsupervised class learning/clustering) is a fundamental challenge in the computational modeling of language acquisition. For example, a plausible model of spoken-language learners must be able to identify the vowel and consonant inventories of the target language solely from the acoustic properties of speech inputs (without reference to text transcriptions, unlike industrial speech recognition systems). This phonetic learning is particularly challenging due to the considerable individual and contextual variations in the acoustic data (Dunbar et al., 2017; Feldman et al., 2009; Vallabha et al., 2007).

In addition to categorizing individual speech sounds (phonemes), language learners must also acquire knowledge of their discrete sequential patterns (phonology). This phonological learning also involves a classification task, since the lexicon of a single language comprises multiple groups of words that adhere to different phonological rules and constraints. For example, nouns and verbs are often governed by separate phonological rules/constraints in various languages (Bobaljik, 1997; Goodenough & Sugita, 1980; Kelly & Bock, 1988; Meyers, 1997; Smith, 1999, 2016). Likewise, semantically distinguished classes of words may also exhibit unique phonological patterns, differing from the rest of the lexicon within the same language; for instance, onomatopoeic expressions (ideophones) in Japanese are formed through the repetition of a bimoraic morpheme (e.g., [kiɾa-kiɾa], meaning “shining”; Ito & Mester, 1995b, 1999), and similar exceptionalities of this word class have been documented across languages (see Dingemanse, 2012, for a review).

Similarly, words of different etymological origins exhibit different phonological patterns. For example, English words are typically categorized according to their Germanic vs. Latinate origins, and this distinction correlates with two different stress patterns found in verbs (Grimshaw, 1985; Grimshaw & Prince, 1986). Etymological classification also provides a crucial framework for analyzing English morphology, as Latinate suffixes predominantly attach to Latinate roots, thereby maintaining etymological consistency throughout entire words (Anshen et al., 1986; Fabb, 1988; O’Donnell, 2015). Comparable etymology-based generalizations have been reported in other languages as well (see the next section for details; Chung, 1983; Fries & Pike, 1949; Lees, 1961; Postal, 1969; Trubetzkoy, 1939/1967; Zimmer, 1969). Furthermore, experimental studies indicate that native speakers possess knowledge of such etymological distinctions, supporting their psychological reality beyond mere statistical observation (Gropen et al., 1989; Moreton & Amano, 1999).

However, modeling the acquisition of such etymology-dependent patterns poses exceptional challenges compared to other class-dependent patterns. The etymological origins of words are not directly observable to general language learners, unlike the abundant syntactic and semantic information embedded in word-sequence data (Brown et al., 2020; Mikolov, Chen, et al., 2013; Mikolov, Sutskever, et al., 2013; Ouyang et al., 2022; Radford et al., 2019). A recent study by Morita and O’Donnell (2022, see also Morita, 2018) introduced a computational framework to investigate the learnability of etymological distinctions in the absence of explicit cues (i.e., without supervision). Case-studying Japanese, they demonstrated that etymological word classes of the language can be inferred solely from phonological information. Specifically, they performed unsupervised clustering on existing Japanese words, represented as strings of phonetic symbols, and their learning model identified word clusters that were significantly well-aligned with the etymologically defined classes. Moreover, the model also recovered the etymology-based phonological generalizations previously described in the literature. These findings offer an empirical justification for linguistic theories that posit etymological distinctions in the mental grammar, by showing that such distinctions need not be acquired through direct access to words’ historical origins (i.e., with supervision), but can instead be inferred from observable phonotactic information.

In the present study, we apply the same clustering algorithm to English words, and demonstrate that the distinction between Germanic and Latinate origins is also learnable from sequential patterns of phonetic symbols appearing in the words (i.e., segmental phonotactics). Our contributions can be summarized as follows.

▪  We present empirical evidence for the unsupervised learnability of the Germanic and Latinate word clusters based solely on phonological information, or segmental phonotactics in particular.
▪  We demonstrate that the identified word clusters recover various linguistic properties of Germanic and Latinate words as proposed in the previous literature.
▪  We highlight several hitherto unnoticed linguistic properties of the discovered word clusters, which can guide future experimental studies.
▪  In conjunction with the findings from the previous study on Japanese (Morita & O’Donnell, 2022), our present study supports the cross-linguistic validity of the proposed learning framework.

The remainder of this paper is organized as follows. In the next section, we commence with a review of related studies concerning etymology-based generalizations of morpho-phonological patterns in English and other languages. We then outline our model for learning etymological classes (or their proxies) exclusively from phonotactic information, alongside an explanation of the dataset employed for the learning simulation. As an outcome of the simulation under these settings, we first report the basic results of the word clustering, including the degree of alignment between the model-detected clusters and the ground-truth etymology. We then examine the linguistic properties of the identified clusters, demonstrating how they recover etymology-based generalizations previously noted in the literature. The final section provides a comprehensive discussion of our findings, outlines potential avenues for future research, and offers concluding remarks.

## BACKGROUND

### Cross-Linguistic Ubiquity of Etymology-Based Phonology

Etymologically-defined lexical subclasses have been extensively documented for various languages, most typically distinguishing between native words and loanwords (see Ito & Mester, 1995b, for reviews). For example:

▪  In Chamorro, mid vowels are absent in native words but present in Spanish loans (Chung, 1983).
▪  In Mohawk, labial consonants [m, b, p] are found in French loans but not in native words (Postal, 1969).
▪  In Mazateco, postnasal stops are systematically voiced in native words but can be voiceless in loans (Fries & Pike, 1949).
▪  German native words do not start with [s], whereas this constraint does not apply to loans (Trubetzkoy, 1939/1967).
▪  In Turkish, high vowels are rounded after labial consonants in native words but not necessarily in loans (Lees, 1961; Zimmer, 1969).

Etymological distinctions are not necessarily binary. For instance, Japanese has ternary distinctions in its morpho-phonology; words are divided into native words, loanwords from Old Chinese, and more recent loans primarily from (Frellesvig, 2010; Fukazawa, 1998; Fukazawa et al., 1998; Gelbart & Kawahara, 2007; Ito & Mester, 1995a, 1995b, 1999, 2003, 2008; Moreton & Amano, 1999; Ota, 2004). Comparable non-binary distinctions have also been reported for other languages, including (Holden, 1976), Turkish (Zimmer, 1985), and Québec French (Hsu & Jesney, 2017, 2018; Paradis & Lebel, 1994).

### Etymology-Based Generalizations in English

Like other languages, English exhibits linguistic generalizations rooted in the Germanic-Latinate distinction. One such generalization involves the etymological consistency of morphemes within a word: Latinate bases tend to be suffixed with Latinate morphemes (Anshen et al., 1986; Fabb, 1988; O’Donnell, 2015). Another well-known example is the difference in stress patterns: Germanic verbs bear initial stress, whereas the initial syllable of Latinate verbs is typically unstressed (Grimshaw, 1985; Grimshaw & Prince, 1986). In addition, velar stops [k] and [g] are subject to “softening” into [s] and [ʤ] respectively in Latinate words (e.g., *electric* → *electricity*), but not in Germanic ones (Chomsky & Halle, 1968; Pierrehumbert, 2006).

The Germanic-Latinate distinction in English also predicts a difference in syntactic patterns of verbs. Most famously, Germanic and Latinate verbs differ in their compatibility with the *double-object construction*. On the one hand, the dative argument of Germanic verbs (e.g., *bring*) can be expressed either as a prepositional phrase or as an indirect object:

▪  ✓*The technician brought the new device to the professor.*
   (prepositional construction)
▪  ✓*The technician brought the professor the new device.*
   (double-object construction)

On the other hand, Latinate verbs are generally considered to disallow the double-object construction (Levin, 1993). For example, the dative argument of the verb *deliver*—despite its semantic similarity to *bring*—can only appear as a prepositional phrase, not as an indirect object:

▪  ✓*The technician delivered the new device to the professor.*
   (prepositional construction)
▪  *The technician delivered the professor the new device.
   (double-object construction)

This syntactic contrast extends to newly coined words as well. Gropen et al. (1989) experimentally demonstrated that the double-object construction was judged more grammatically acceptable with quasi-Germanic (monosyllabic) nonce verbs than with quasi-Latinate (polysyllabic) nonce verbs.

As a caveat, there are exceptions to this etymology-based generalization. For instance, *pass* is a Latinate verb that nonetheless permits the double-object construction:

▪  ✓*The technician passed the new device to the professor.*
   (prepositional construction)
▪  ✓*The technician passed the professor the new device.*
   (double-object construction)

Interestingly, our learning model—introduced in the next section—“correctly misclassified” such exceptions into the Germanic-like word cluster on the basis of their phonotactic Germanicity, thereby outperforming grammaticality predictions based on true etymological origin.

## MATERIALS AND METHODS

This section provides a high-level explanation of our learning model for inducing word clusters corresponding to etymological distinctions, and the dataset used to train it.

### Overview of the Learning Model

We model the learning of etymological lexical classes in the framework of unsupervised word clustering. The learning model receives English words—represented as strings of phonetic symbols—as its inputs and groups them into an optimal number of clusters following a certain statistical policy (explained below). Most importantly, the model has no access to ground-truth etymological information (such as “Germanic origin” or “Latinate origin”) during its learning process, making it *unsupervised*. This setup more closely approximates human language acquisition than approaches rely on access to ground-truth etymology (i.e., supervised learning).

Our approach to word clustering is grounded in Bayesian inference (Anderson, 1990; Tenenbaum, 1999): We define a prior probability of word partitions (i.e., learning hypotheses) as well as their likelihood with respect to the data. Then, the optimal clustering is determined by the maximization of the posterior probability, which is proportional to the product of the prior and the likelihood.

In the remainder of this section, we focus on a high-level explanation of the model components, abstracting away from mathematical details. Interested readers are referred to Morita and O’Donnell (2022). In addition, the Python code used for this study is publicly available in the public repository.^1^ The values of hyperparameters are reported in Appendix A.

#### Prior.

We adopt a non-parametric prior distribution on cluster assignments known as the *Dirichlet process* (DP; Antoniak, 1974; Ferguson, 1973; Sethuraman, 1994). The DP prior prioritizes grouping words into the fewest possible clusters; it assigns a geometrically smaller probability (in expectation) to assignments spread over a greater number of hypothesized clusters. In linguistics and other fields, the DP has been widely used as a prior over lexica and other similar inventories (e.g., Anderson, 1990; Feldman et al., 2013; Goldwater et al., 2006, 2009; Kemp et al., 2007; O’Donnell, 2015; Teh, 2006; Teh et al., 2006).

#### Likelihood.

We assume that each word is sampled from a probability distribution whose parameters are associated with the cluster to which the word belongs.^2^ Then, the likelihood of a word partition is defined by the product of the probabilities of all words in the dataset given their cluster assignments defined by the partition.

Clusters with fewer words have less phonotactic variability and thus the probability distribution associated with the cluster is able to be more sharply peaked around a smaller set of phonotactic patterns assigning a higher probability to each word. For this reason, the likelihood favors finer-grained word partitions; in an extreme case, the likelihood is maximized when each individual word forms its own cluster, specialized to generate just that particular word. Thus, the likelihood and the prior have opposing preferences for word partitions, and learning amounts to balancing a tradeoff between these opposing pressures.

For the specific implementation of the likelihood, we utilize a trigram model of phoneme strings (with hierarchical backed-off smoothing; Goldwater et al., 2006; Teh, 2006). This model defines probabilities of phonemes conditioned on their two closest predecessors within words, and the overall word probability is the product of these phoneme probabilities. While trigram models may not capture all phonotactic patterns in the world’s languages (Hansson, 2001; Heinz, 2010; Rose & Walker, 2004),^3^ they can effectively express local phonotactic dependencies among segments and account for a major portion of attested phonotactics (Gafos, 1999; Hayes & Wilson, 2008; Ní Chiosáin & Padgett, 2001). With this model, the likelihood of a word partition is simply the product of the probabilities of all words in the dataset given their cluster assignments defined by the partition.

#### Bayesian Inference.

The optimal word clustering is formalized as the computation of the *posterior* probability, proportional to the product of the prior and likelihood by Bayes’ theorem. A similar approach to balancing between simplicity and fit to the data is inherent in various theories of inductive inference (Bernardo & Smith, 1994; Clark & Lappin, 2011; Grünwald, 2007; Jain et al., 1999; Li & Vitányi, 2008; Rissanen & Ristad, 1994; Shalev-Shwartz & Ben-David, 2014; Vapnik, 1998).

A major challenge in this Bayesian inference is that the exact computation of the posterior probabilities is typically computationally intractable. Accordingly, we resort to variational approximation (specifically, the mean-field approximation) of the posterior to effectively handle the computational complexity and obtain practical solutions (Bishop, 2006; Blei & Jordan, 2006; Blei et al., 2017; Wainwright & Jordan, 2008; Wang et al., 2011).

### Data

The clustering method introduced above was applied to the (British) English portion of the CELEX lexical database (Baayen et al., 1995). We adopted the original phonetic transcription of the dataset (called DISC; see Appendix B for details) to represent the input.

Our model solely relies on the segmental information in words; accordingly, prosodic information—specifically, stress and syllable boundary markers—was removed from the transcriptions.^4^

Our data only distinguished lemmas, ignoring spelling and inflectional variations among words (such as singular-plural distinctions).^5^ We further limited the data to lemmas with positive frequency in the Collins Birmingham University International Language Database (COBUILD; Sinclair, 1987). After filtering, there remained 38,731 words in the input data.

## CLUSTERING RESULTS

The unsupervised clustering revealed two primary sublexical clusters, labeled as Sublex≈_Germanic_ and Sublex≈_Latinate_ (Table 1). Additionally, a small cluster, labeled as Sublex≈_-ity_, was identified, consisting of words that end with the suffix *-ity*.^6^ The emergence of this minor cluster suggests that a significant proportion of English words are formed through the suffixation of *-ity*, indicating the exceptional productivity of this suffix. Given that our model operates without awareness of morphological structures, there remains little else to discuss on Sublex≈_-ity_. Therefore, the remainder of this paper is devoted to discussing linguistic properties of the other two major clusters, Sublex≈_Germanic_ and Sublex≈_Latinate_.

**Table 1.** Clustering results based on the maximum-a-posteriori (MAP) prediction of the model.

Cluster Name	#Words	Proportion
Sublex≈_Germanic_	23,354	60.3%
Sublex≈_Latinate_	15,174	39.2%
Sublex≈_-ity_	203	0.5%

**Figure 1.**
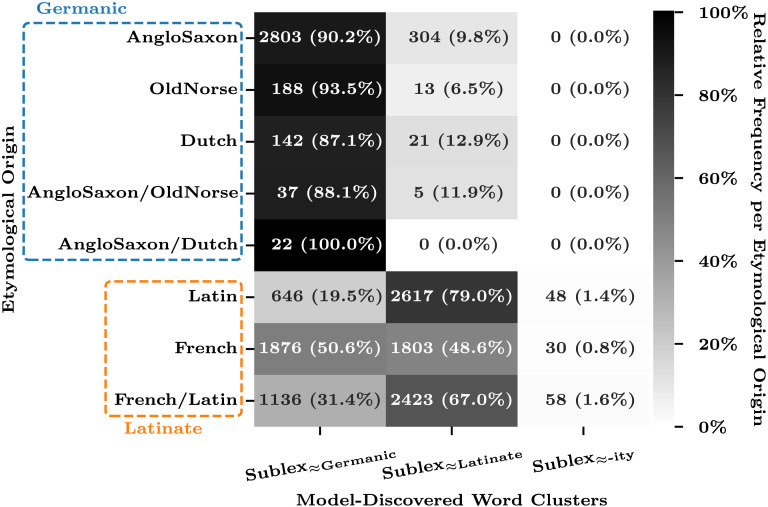
Alignment between the model-discovered clusters (columns, MAP classification) and the etymological origin according to Wikipedia (rows).

Figure 1 illustrates the alignment between the discovered clusters (columns) and ground-truth etymological origins (rows). Due to the absence of etymological information in the CELEX dataset, we evaluated only a subset of the data (3,535 Germanic words and 10,637 Latinate words) whose etymological origin was identified in Wikipedia articles (see Appendix C for details). Each cell of the heatmap is annotated with the word counts of the corresponding cluster-etymology intersection, followed by their relative frequency (in parentheses) per etymological origin (i.e., normalized over the columns, per row). The heatmap darkness also represents this relative frequency. The etymological origins are grouped into Germanic and Latinate by blue and orange dashed lines, respectively. The rows labeled with multiple origins (e.g., “AngloSaxon/OldNorse”) represent the words duplicated in the Wikipedia lists of the corresponding origins.

Germanic words—of Anglo-Saxon, Old Norse, or Dutch origin—were closely aligned with the discovered Sublex≈_Germanic_ class. By contrast, the alignment between the discovered Sublex≈_Latinate_ class and words of etymologically Latinate origin showed less consistency; while Latin-derived words predominantly clustered into Sublex≈_Latinate_, those of French origin were almost evenly split between the two clusters. However, this imperfect alignment of the model predictions with the ground-truth etymology is not necessarily a disappointing result; later in this work, we will see that our model’s “misclassifications” exhibit stronger correlations with the grammaticality of double-object constructions than the ground-truth etymology, thus providing a more effective account of the “exceptions” in the previous generalizations.

To quantitatively assess the significance of the alignment between our unsupervised clustering and the ground-truth etymology, we employed the V-measure metric (Roseberg & Hirschberg, 2007). The V-measure evaluates the similarity between predicted clustering and ground-truth classification based on two desiderata:

▪  Homogeneity: each of the predicted clusters should contain only members of a single ground-truth class.
▪  Completeness: the members of each ground-truth class should be grouped into the same cluster.

Homogeneity and completeness are formally defined based on a normalized variant of the conditional entropy, both falling on a scale of 0 (worst) to 1 (best). The V-measure score is their harmonic mean (analogous to the F1-score).

The V-measure score of our clustering result was 0.198, significantly greater than the chance-level baseline drawn by random shuffling of the ground-truth word origins (*p* < 10^−5^).^7^

## PHONOTACTIC CHARACTERIZATION OF THE DISCOVERED WORD CLUSTERS

In this section, we investigate the phonotactic properties of the model-detected clusters, Sublex≈_Germanic_ and Sublex≈_Latinate_, examining if they are consistent with the observations made in the previous literature. We also conduct a data-driven exploration of the phonotactic features of these clusters, aiming to uncover previously unrecognized patterns.

### Metric of Representativeness

Following Morita and O’Donnell (2022), our analysis of the phonotactic properties of the identified clusters is grounded in a metric of *representativeness* of phonetic segments (Tenenbaum & Griffiths, 2001). This metric assesses the relative likelihood that a sequence of phonetic segments comes from a particular cluster compared to the other(s). Essentially, representativeness is highest for patterns that are highly probable in the target cluster but improbable in the other(s). Consequently, it helps us identify (strings of) segments that provide informative cues for classification.

Formally, the representativeness *R*(**x**, *k*) of a string of phonetic segments **x** ≔ (*x*_1_, …, *x*_*m*_) with respect to the word cluster *k* is defined by the logarithmic ratio of the posterior predictive probability of **x** appearing somewhere in a word belonging to the cluster *k*, to the average posterior predictive probability of **x** over all other clusters:

$$R\left(x,k\right)≔\log\frac{p\left(\ldots x\ldots |k,d\right)}{\sum_{k^{\prime }\neq k}p\left(\ldots x\ldots |k^{\prime },d\right)p\left(k^{\prime }|d,k^{\prime }\neq k\right)}$$ (1)

where **d** denotes the training data of the clustering. For a detailed explanation of how the representativeness is computed, we refer interested readers to Morita and O’Donnell (2022).

### Recovery of Previous Generalization Regarding Stress Patterns

We initiate our phonotactic analyses by showing that the model recovers generalizations proposed in the previous literature. Specifically, we discuss the prosodic characterization that Germanic verbs bear initial stress whereas Latinate verbs have unstressed initial syllable (Grimshaw, 1985; Grimshaw & Prince, 1986).

It is important to note that the training data for our model did not explicitly include prosodic information of words, such as syllables or stress patterns. Thus, the model cannot make direct predictions regarding the stress patterns of word-initial syllables. Nonetheless, the model can still make indirect predictions for the prosodic patterns, exploiting the fact that most of the English vowels are reduced to schwa [ә] in unstressed positions. Specifically, we can evaluate which English vowels are most representative of Sublex≈_Latinate_ and Sublex≈_Germanic_ when they appear as the first vowel in a word. If schwa has a high degree of representativeness with respect to Sublex≈_Latinate_ in first position, it indicates that the unstressed word-initial syllable is representative of the word cluster.

We focus our analysis on the initial vowels of polysyllabic words,^8^ simply because monosyllabic words always bear initial stress. Moreover, Germanic words are more likely to be monosyllabic than Latinate words (Gropen et al., 1989); thus, without the requirement of polysyllabicity, initial unstressed vowels can be representative of Sublex≈_Latinate_ merely due to the greater expected word length, derailing our primary interest in stress patterns.^9^

Table 2 reports the representativeness scores of all English vowels with respect to Sublex≈_Germanic_ and Sublex≈_Latinate_ when occurring in the first syllable of polysyllabic words.^10^ The phonetic transcription (of British English) was translated from DISC to IPA for readability.

**Table 2.** Representativeness of the first vowels in polysyllabic words (with zero to three initial consonants and zero to five internal consonants between the first and second vowels) with respect to Sublex≈_Germanic_ and Sublex≈_Latinate_.

Vowel	Sublex≈_Germanic_	Sublex≈_Latinate_
ә	−1.832312	1.720911
Ι	−1.173901	1.136778
Ʊә	−0.819426	0.736090
Ιә	−0.704950	0.691387
ɛ	−0.291054	0.292225
æ	−0.162233	0.160080
ɒ	−0.127456	0.135075
ᾶː	−0.470889	−0.043847
ɜː	0.063449	−0.074478
æ˜	−0.570518	−0.184845
uː	0.343961	−0.341091
ɔː	0.342331	−0.342073
æ˜ː	−0.314203	−0.396665
ɒ˜ː	−0.277864	−0.426937
aΙ	0.529269	−0.534647
Ʌ	0.545830	−0.537470
әƱ	0.584508	−0.576595
ɔΙ	0.555049	−0.587985
αː	0.608208	−0.597709
iː	0.849564	−0.840888
Ʊ	1.001237	−0.998456
ɛә	1.141281	−1.181892
eΙ	1.172851	−1.183918
aƱ	1.697911	−1.707058

Schwa [ә] scored the lowest in Sublex≈_Germanic_ and the highest in Sublex≈_Latinate_. This finding aligns with the previous generalization that initial stress (i.e., non-schwa initial vowels) is a hallmark of Germanic words.

### Data-Driven Investigation of Representative Phonotactic Patterns

In addition to recovering the previous generalization of Germanic and Latinate phonology, our model can also be used for a data-driven investigation to identify class-specific phonotactic patterns. Specifically, ranking phonotactic patterns by their representativeness with respect to each cluster can unveil previously unnoticed characteristic patterns, suggesting new hypotheses for future experimental studies.

It is important to note that the representativeness-based analysis does not eliminate ungrammatical phonotactic patterns that never appear in English words. This is because our trigram phonotactic model is smoothed and assigns non-zero probabilities to unobserved patterns; consequently, a string of segments that is extremely improbable across all clusters can still be representative of one cluster if its probability in that cluster is relatively greater than in the others. Given the challenge of interpreting such ungrammatical (and rare) patterns, we limit our ranking to substrings with a minimum frequency of ten (cf. Morita & O’Donnell, 2022).

Table 3 presents the top-five uni- to trigram substrings ranked by their representativeness.^11^ These substrings are largely consistent with our intuition. For instance, many of the high-ranking bigrams and trigrams for Sublex≈_Latinate_ correspond to the Latinate suffix *-(at)ion*, as exemplified by [ʒ n̩] and [eΙ ʃ n̩].

**Table 3.** Uni- to trigram substrings yielding the greatest representativeness. The non-IPA tokens, END and * (asterisk), represent the word-final position and linking *r*, respectively.

	Rank	Sublex≈_Germanic_					Sublex≈_Latinate_				
		Substring			Rep. Score	Examples	Substring			Rep. Score	Examples
Unigram	1	ð			1.5379	*bathe*, *mother*	n̩			1.9389	*essence*, *nation*
	2	aƱ			1.4040	*about*, *loud*	Ʊә			1.4407	*cure*, *duration*
	3	w			1.3100	*work*, *wound*	j			1.2592	*accuse*, *use*
	4	ŋ			1.2497	*blink*, *swing*	Ιә			0.9589	*rear*, *serial*
	5	h			1.2479	*hand*, *hole*	v			0.8201	*survive*, *vacation*
Bigram	1	h	uː		4.7799	*hoop*, *who*	ʃ	n̩		5.5336	*ocean*, *sufficient*
	2	h	Ʊ		4.7651	*hook*, *likelihood*	tʃ	Ʊә		4.4053	*actual*, *virtuous*
	3	f	Ʊ		4.3186	*careful*, *foot*	eΙ	ʃ		4.3633	*facial*, *ratio*
	4	ɛә	*		4.1726	*hair*, *there*	j	Ʊ		3.8582	*occupy*, *popular*
	5	r	aƱ		4.0835	*brown*, *ground*	ʒ	n̩		3.7467	*decision*, *vision*
Trigram	1	ŋ	ɡ	l̩	9.0861	*mingle*, *wrangle*	eΙ	ʃ	n̩	8.8990	*education*, *patient*
	2	h	aƱ	s	8.1806	*house*, *warehouse*	j	Ʊ	r	8.6861	*accurate*, *mercury*
	3	iː	p	END	7.8941	*deep*, *sleep*	p	tʃ	Ʊә	7.8682	*conceptual*, *sumptuous*
	4	Ʌ	ʃ	END	7.8941	*flush*, *rush*	k	ʃ	n̩	7.5597	*action*, *section*
	5	k	Ʊ	k	7.3461	*cook*, *cookie*	ʃ	n̩	ә	7.5533	*stationary*, *nationalism*

Similarly, it is reasonable that the word final [iː p] (represented as a trigram [iː p END] in our model) is characteristic of Sublex≈_Germanic_, given that most words exhibiting this phonotactic pattern are Germanic, such as *creep*, *deep*, *heap*, *leap*, *sleep*, *sheep*, *steep*, and *sweep* (Stevenson & Lindberg, 2010).^12^ Likewise, the most representative bigram [h uː] appears exclusively in Germanic words like *hoop* and *who(m)*. Despite their plausibility, however, these phonetic characterizations of Germanic words had never been documented previously to our knowledge, indicating the effectiveness of data-driven investigation for identifying novel patterns.

## WORD-INTERNAL CONSISTENCY OF MORPHEME ETYMOLOGY

In the previous section, we phonotactically characterized the word clusters identified by our model. Conversely, our model infers these clusters based on the word-internal correlations among such phonotactic patterns; frequent cooccurrences of different substrings within words are better explained by a mixture of multiple distributions—each fitting to specific cooccurring patterns—rather than by a single trigram distribution, which assumes i.i.d. sampling (or mere coincidence) of the cooccurring substrings. Consequently, the presence of long words is essential for our model to observe sufficient cooccurrences.

Long words are typically formed through the affixation of morphemes. Therefore, successful learning of word clusters presupposes the word-internal consistency of phonotactic distributions across morphemes. In other words, our model exploits the etymological consistency across morphemes (e.g., Latinate suffixes attach to Latinate bases; Anshen et al., 1986; Fabb, 1988; O’Donnell, 2015). In this section, we demonstrate that this word-internal etymological consistency is indeed reflected in our model, by showing that it classifies different base morphemes of a common affix into the same cluster.

**Figure 2.**
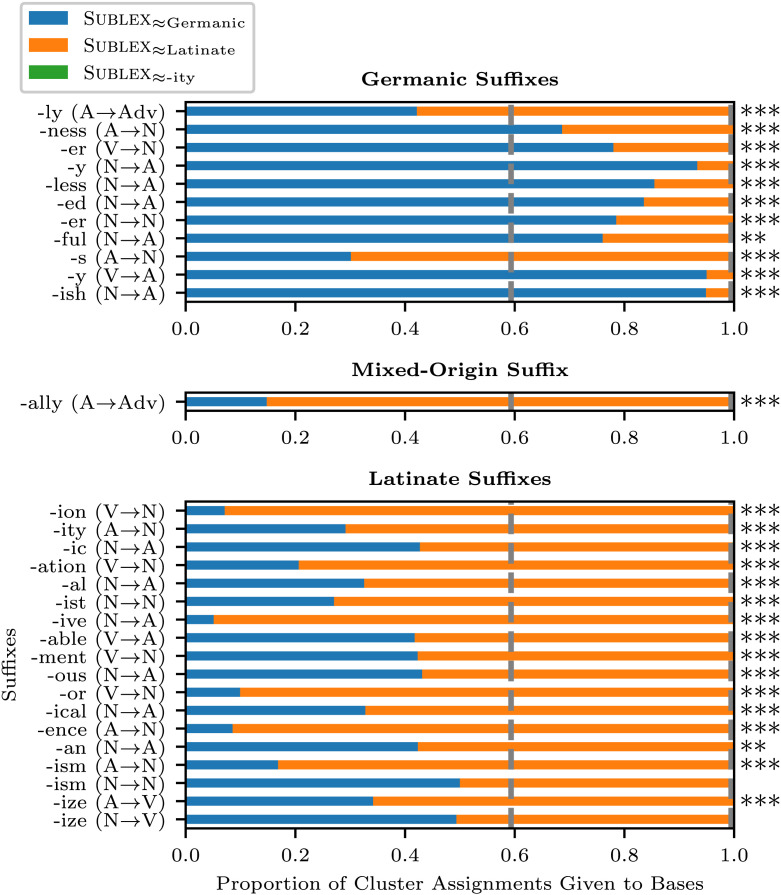
The proportion of the MAP cluster assignments given to the bases of the top thirty type-frequent suffixes.

Figure 2 illustrates the proportion of cluster assignments given to the bases of the thirty most productive suffixes (e.g., the bases of *-ion* include *execute*, *suspend*, *profess*, etc.), ranked by the number of words derived through the suffixation (i.e., type frequency), as documented in the CELEX dataset. The vertical dashed lines indicate the proportion of the cluster assignments expected from the overall ratio (i.e., under the null hypothesis; Sublex≈_Germanic_: 59.33%, Sublex≈_Latinate_: 40.13%, Sublex≈_-ty_: 0.53%). The asterisks on the right to the bars represent the statistical significance of the deviation from the null hypothesis according to the multinomial test (*: *p* < 0.05, **: *p* < 0.01, ***: *p* < 0.001). The conversion of the grammatical category by the suffixation is annotated in the parentheses (e.g., “A → Adv” represents derivation of adjectives to adverbs). The suffix *-ally*, identified as a single suffix in the CELEX, was parsed as a concatenation of Latinate (*-al*) and Germanic (*-ly*) suffixes, and thus labeled as “Mixed-Origin”.

Eleven of these suffixes were of Germanic origin, and eighteen were of Latinate origin (Stevenson & Lindberg, 2010); the remaining suffix, *-ally*, was analyzed as the concatenation of the Latinate suffix *-al* and the Germanic suffix *-ly*—although the CELEX treats it as a single morpheme—and thus, it was categorized separately as a “mixed-origin” suffix. The suffixes and their corresponding bases were identified according to the morphological structures provided in the CELEX, with the clustering based on the freestanding forms of the bases, which were also identified in the CELEX (otherwise excluded from the analysis).

The vertical dashed lines in the figure indicate the overall proportion of cluster assignments over the entire CELEX dataset (Sublex≈_Germanic_: 59.33%, Sublex≈_Latinate_: 40.13%, Sublex≈_-ty_: 0.53%). Using this overall ratio as the null-hypothetical parameters of the multinomial test,^13^ we assessed the statistical significance of the suffix-dependent tendencies of base clustering towards Sublex≈_Germanic_ or Sublex≈_Latinate_.

Overall, our model systematically classified the bases of Germanic and Latinate suffixes into Sublex≈_Germanic_ and Sublex≈_Latinate_, respectively, thereby recovering the generalizations documented in the previous literature with statistical significance (Anshen et al., 1986; Fabb, 1988; O’Donnell, 2015). Exceptions were observed for the bases of the Germanic suffixes *-ly* (e.g., *slow* → *slowly*) and *-s*^14^ (e.g., *acoustic* → *acoustics*, *odd* → *odds*), which tended to be classified into Sublex≈_Latinate_. In addition, two Latinate suffixes, *-ism* (for noun-to-noun derivation) and *-ize* (for noun-to-verb derivation), did not exhibit a statistically significant deviation from the null-hypothetical clustering ratio.

## SYNTACTIC PREDICTIONS FROM THE CLUSTERING RESULTS

Finally, we assess our model’s capability to predict the syntactic grammaticality of double-object constructions (hereinafter abbreviated as DOC) of dative verbs. It should be noted that DOC can be ungrammatical for various reasons; for example, DOC is not permitted with verbs that have certain types of semantics, such as communication of propositions and propositional attitudes (e.g., *announce*, *report*) and manner of speaking (e.g., *scream*, *whisper*; Levin, 1993). The influence of verb semantics is further supported by an experimental study by Gropen et al. (1989), which showed that nonce verbs denoting transfer of possession were judged more acceptable for DOC than those with other meanings. Beyond the lexical properties of individual verbs, DOC acceptability is also influenced by contextual and discourse factors, including the animacy, novelty, and length of the dative and accusative arguments (Bresnan, 2007; Bresnan et al., 2007; Collins, 1995; Rathi et al., 2024; Sinclair et al., 2022).

Again, our model relies purely on phonotactic information and cannot make any syntactic predictions per se. However, our model can indirectly infer the grammaticality by clustering dative verbs based on phonotactics and substituting the etymology-based generalizations with these model-detected clusters (cf. Gropen et al., 1989).

Specifically, we evaluate the alignment of our model’s clustering with the following distinction:

▪  ✓DOC verbs:
   Dative verbs that permit DOC (and the prepositional construction).
▪  *DOC-Lat verbs:
   Dative verbs that are said to disallow DOC *solely due to their Latinateness* (i.e., no other factors can distinguish them from ✓DOC verbs; Levin, 1993).

The test data for this assessment were derived from Levin (1993).^15^^,^^16^ A critical aspect of these data is that not all of ✓DOC verbs are etymologically Germanic in fact; *there are exceptional Latinate verbs that allow DOC*.^17^ This empirical fact gives room for our model—by predicting these exceptions—to outperform the etymology-based generalization in the previous literature.

**Figure 3.**
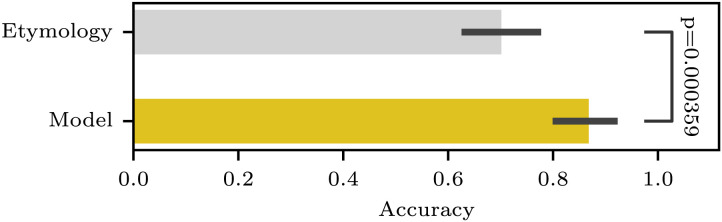
Accuracy scores of DOC grammatical prediction by the phonotactics-based clustering and the ground-truth etymology.

Figure 3 reports the accuracy of distinguishing ✓DOC from *DOC-Lat verbs based on our clustering results and the true etymological classifications. Error bars indicate 95% confidence intervals, estimated from 1000 bootstrap samples. The statistical significance of the difference between the model- and etymology-based predictions was assessed using the exact McNemar’s test.

**Figure 4.**
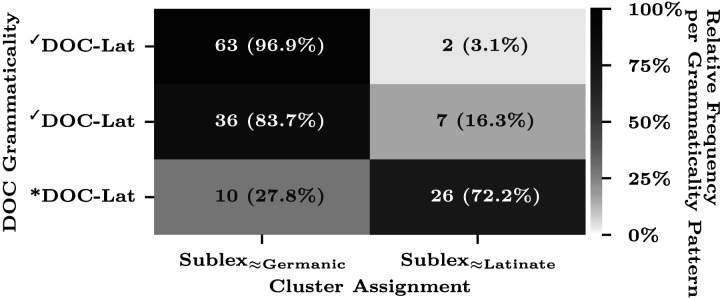
Alignment between the model-discovered clusters (columns, MAP classification) and the DOC grammaticality patterns (rows). Each cell of the heatmap is annotated with the word counts of the corresponding cluster-grammaticality intersection, followed by their relative frequency (in parentheses) per grammaticality pattern (i.e., normalized over the columns, per row). The heatmap darkness also represents this relative frequency.

Remarkably, the model predictions (0.8681) surpassed those based on the true etymology (0.7014). As already noted above, this advantage is due to the fact that some of the Latinate verbs exceptionally permit DOC (termed ✓DOC-Lat hereinafter to distinguish them from the non-Latinate verbs, termed ✓DOC-Lat) and our model “correctly misclassified” these exceptions to Sublex≈_Germanic_ based on their phonotactic patterns (Figure 4; see Appendix D for the clustering results of individual verbs). This outcome suggests that the grammaticality of DOC is more accurately generalized by phonotactic patterns rather than by the etymological origins. Indeed, this finding aligns with the experimental study by Gropen et al. (1989), who showed the productivity of DOC utilizing monosyllabic and polysyllabic nonce words that characterized Germanic and Latinate verbs, respectively.

## GENERAL DISCUSSION

### Findings and Implications

In this study, we demonstrated that the Germanic-Latinate distinction within the English lexicon is learnable from phonotactic information alone, without supervision. Specifically, we showed that the model-discovered clusters:

▪  Broadly aligned with the ground-truth etymological classification,
▪  Recovered known phonotactic generalizations (stress patterns),
▪  Revealed hitherto unnoticed phonotactic properties (e.g., [iːp] and [huː] as strong cues to membership in the Germanic-like cluster),
▪  Captured the etymological consistency of morphemes within words, and
▪  Predicted the grammaticality of DOC more accurately than the ground-truth etymology.

By empirically demonstrating the learnability of Latinate- and Germanic-like word clusters, the present study supports the psychological reality of linguistic theories that posit an etymological distinction in the mental grammar of native speakers (e.g., Chomsky & Halle, 1968; Ito & Mester, 1995b). Even though language learners do not have access to information about words’ historical origins, they can acquire word clusters that approximate this distinction solely from phonotactic data. Moreover, the clusters identified by our model can capture linguistically meaningful generalizations more effectively, as evidenced by their superior performance in predicting DOC grammaticality.

The model predictions also offer opportunities for further experimental investigation into class-dependent linguistic properties. For example, researchers could test the psychological reality of the uni- to trigrams that our model identified as representative of the Latinate- and Germanic-like word clusters, employing experimental paradigms similar to those used in the previous studies (e.g., Moreton & Amano, 1999). Similarly, the clustering results could be leveraged to experimentally examine predictions about DOC grammaticality derived from our model (cf. Gropen et al., 1989).

Finally, our findings support the cross-linguistic applicability of the proposed learning framework. Morita and O’Donnell (2022) demonstrated that the etymological word classes in Japanese can also be learned from phonotactic information using the same model, despite prior claims that such learning depends on orthographic cues unique to Japanese (i.e., distinct writing systems are used for native vs. loanwords; Gelbart & Kawahara, 2007). Because the phonotactics-based approach requires only phonetic transcriptions, it can be applied to any spoken language, paving the way for future cross-linguistic tests of its universality.

Beyond cognitive-scientific inquiry, phonotactics-based clustering may also serve as a useful tool in historical linguistics, providing a starting point for identifying word etymology prior to more detailed analyses. It should be noted, however, that the method is entirely unsupervised; that is, alignments between the resulting clusters and true etymological classes are not guided by any supervision and therefore not guaranteed, as partially demonstrated by our DOC analysis. Conversely, the possibility of such misalignments raises an intriguing question for future research: are there languages in which unsupervised clustering would reveal other kinds of word classes instead of etymological ones? For example, the grammatical gender of French nouns is largely predictable from their phonotactic patterns (at least for a supervised classifier; Lyster, 2006), and thus an unsupervised learner might discover gender-like clusters rather than etymology-like ones in such languages. We therefore expect the model to recover whichever lexical distinction is most strongly cued by phonotactics in a given language, rather than privileging etymology per se. Relatedly, our proposed model cannot represent multiple, partially overlapping lexical distinctions (e.g., etymology- and gender-based distinctions simultaneously). A possible extension to address this limitation would be to replace the DP with an Indian buffet process (Griffiths & Ghahramani, 2006, 2011), which allows words to have probabilistic membership in multiple latent classes.

### Alternative Approaches to Sublexicon Learning

This study adopted a Bayesian framework to demonstrate the learnability of sublexical word clusters in the absence of supervision (Morita & O’Donnell, 2022). This section reviews alternative approaches to related problems explored in previous work.

Ito and Mester (1999) argued that the linguistic input available at an early stage of language acquisition may be restricted to words belonging to a specific sublexical class—particularly the native sublexicon—thereby leading learners to acquire phonotactic knowledge specialized for that class. Subsequently, learners may come to recognize the existence of additional sublexica when they encounter exceptions to this initially acquired phonotactic grammar. The plausibility of this staged-input hypothesis remains controversial. On the one hand, Gropen et al. (1989) observed that Latinate dative verbs were absent from spontaneous utterances of English-speaking children and were also rare in child-directed speech (both in either DOC and prepositional constructions). On the other hand, Ota (1999, 2003, 2004) reported that more than 50% of the words produced by two-year-old Japanese children exhibited phonotactic patterns characteristic of non-native sublexica.

Pater (2005) proposed a learning algorithm that identifies words exhibiting exceptional phonotactic patterns and classifies them into a separate sublexicon from other, non-exceptional words. Pater tested this algorithm only on idealized toy data—consisting of nine hypothetical words—and it has not been evaluated on an empirical dataset (see Morita & O’Donnell, 2022, for a more detailed discussion).

Becker and Gouskova (2016) offered a probabilistic account of sublexicon-specific morpho-phonological patterns in Russian. They posited a classifier that groups masculine nominative nouns into two classes based on their phonotactic structure and predicts whether vowel deletion applies in their genitive forms according to the classification. This approach aligns with our analysis of DOC-possibility, abstracting away from the specific probability distributions assumed. For training, however, Becker and Gouskova’s sublexical classifier requires either supervision or explicit cues indicating the desired classification (cf. Ito et al., 2001). In their case study of Russian, the nominative and genitive forms of each noun are assumed to be pre-identified, allowing the learner to infer the existence of two sublexica from the applicability of vowel deletion in the genitives. By contrast, our Bayesian method requires only sequences of phonetic segments as input data and can therefore be applied to any spoken language.

### Limitations and Possible Extensions of the Learning Model

We adopted an *n*-gram model of phonotactics to evaluate the likelihood of sublexical clusters. While this model can exhaustively capture local phonotactic dependencies among *n* adjacent segments—accounting for a substantial portion of attested patterns (Gafos, 1999; Hayes & Wilson, 2008; Ní Chiosáin & Padgett, 2001)—some languages exhibit unbounded long-distance dependencies (e.g., anteriority agreement between sibilants in Sapir & Hoijer, 1967).

To accommodate such long-distance dependencies, the Bayesian framework for sublexicon learning can be extended by incorporating a more expressive phonotactic model. For instance, Futrell et al. (2017) extended the *n*-gram model to capture distant dependencies between segments that share phonological features. Similarly, our learning paradigm is compatible with Optimality-Theoretic models, provided that they define a probability distribution over word forms (Daland et al., 2011; Hayes & Wilson, 2008).^18^

Long-distance dependencies can also be modeled using modern neural-network architectures (Gu & Dao, 2024; Vaswani et al., 2017). However, these models permit unrestricted interactions among arbitrary phonemes, which conflicts with cross-linguistic evidence that only phonetically related or similar segments interact at a distance (Hayes & Wilson, 2008; Morita & O’Donnell, 2022). For this reason, feature-based models constitute a more linguistically plausible direction for extending the present framework.

Feature-based models may also identify sublexical differences in local dependencies more efficiently. While the *n*-gram model treats individual segments as arbitrary symbols, feature-based models exploit inherent phonetic similarities and differences among segments (see Futrell et al. 2017, for an empirical comparison).^19^

Another limitation of our study is its lack of prosodic representations (Kager & Pater, 2012). Although our model indirectly captured the difference in stress patterns between Germanic and Latinate verbs by exploiting segmental alternations associated with stress (vowel reduction), this strategy would not generalize to other languages. The extended *n*-gram model proposed by Futrell et al. (2017) addresses this issue by encoding prosodic information as suprasegmental features of vowels. Alternatively, one might adopt a hierarchical generative model that represents latent prosodic structures (Lee et al., 2015).

It should be noted, however, that all of the potential extensions discussed above are computationally more demanding than the *n*-gram model. This issue is particularly acute in the context of word-cluster learning, where multiple phonotactic models must be instantiated, one for each cluster. Indeed, even our *n*-gram model required a dedicated approximation method for Bayesian inference (variational inference; Bishop, 2006; Blei & Jordan, 2006; Blei et al., 2017; Wainwright & Jordan, 2008; Wang et al., 2011) to make full-scale analysis of the entire lexicon computationally feasible,^20^ Because this approximation method was specifically tailored to the *n*-gram phonotactic model, adapting it to other phonotactic models requires customized formulations of the approximate posterior, which is technically non-trivial and thus poses a major challenge for future investigations of more advanced phonotactic models.

Beyond the model design and data representation, the full-batch learning paradigm adopted in this study—assuming that the learner has simultaneous access to the entire English vocabulary—is cognitively unrealistic. In reality, children acquire their vocabulary incrementally, and it remains an open question how word clusters might be identified under such an online learning scenario. In particular, the rarity of words from certain sublexica in child-directed speech may hinder the statistical inference of the corresponding word clusters (e.g., Latinate words in English; Gropen et al., 1989).

## ACKNOWLEDGMENTS

We gratefully acknowledge the support of the Canada CIFAR AI Chairs Program and the Natural Sciences and Engineering Research Council of Canada (NSERC).

## FUNDING INFORMATION

This study was supported by JST AIP Accelerated Program (JPMJCR25U6), ACT-X (JPMJAX21AN), and CREST (JPMJCR22P5); JSPS Grant-in-Aid for Early-Career Scientists (JP21K17805) and for Scientific Research A (JP24H00774), B (JP22H03914), and C (JP24K15087); and Kayamori Foundation of Informational Science Advancement (K35XXVIII620).

## AUTHOR CONTRIBUTIONS

TM and TJO jointly contributed to the conceptualization and writing of this study. TM implemented the learning simulation and performed the analyses. TJO supervised this project.

## Notes

^1^ https://github.com/tkc-morita/variational_inference_DP_mix_HDP_topic_ngram.^2^ The parameters of each word cluster are optimized during the learning process together with the cluster assignments.^3^ A higher likelihood might be achieved by employing an artificial neural network capable of modeling longer-range stochastic dependencies on preceding elements. Indeed, the current state-of-the-art model for time series data, Transformer, can be mathematically interpreted as an extended version of the *n*-gram model, allowing a substantially larger effective Markov order of *n* − 1 (Vaswani et al., 2017). However, permitting long-distance dependencies among arbitrary segments is not appropriate for modeling phonotactics, since cross-linguistically, only phonetically related or similar sounds interact at a distance (Hayes & Wilson, 2008; Morita & O’Donnell, 2022, see General Discussion for more details). Furthermore, our simpler trigram model integrates more readily with the DP prior used in our Bayesian inference framework.^4^ There are several possibilities for extending our trigram model to incorporate prosodic information of words. See the Limitations section for details.^5^ Some lemmas had more than one possible pronunciation; in such cases, we adopted the first (leftmost) entry.^6^ All but one of the 203 words in Sublex≈_−ity_ were singular, with a single exception of *susceptibilities*.^7^ The *p*-value was estimated using Monte Carlo: We sampled 100,000 random permutations of the ground-truth classifications, and the *p*-value was defined by the proportion of the random permutations whose V-measure score was greater than the model performance (Ewens, 2003). None of the 100,000 random permutations achieved a V-measure as great as the model, thus yielding *p* < 10^−5^.^8^ To compute the representativeness of a vowel V_1_ appearing in the first syllable of a polysyllabic word, we replace *p*(… **x** … | *k*^(′)^, **d**) in Eq. 1 with *p*(V_1_ | C_1_*__C_2_*V_2_, *k*^(′)^, **d**): that is, the posterior predictive probability of V_1_ conditioned on the context C_1_*__C_2_*V_2_, where C_1_* and C_2_* represent the existence of an arbitrary number of consonants (including “no consonants”) in the positions and V_2_ represents the existence of another vowel in the word. In practice, we constrain C_1_* up to three consonants and C_2_* up to five consonants, based on the maximum length of the word-initial and internal consonant clusters in the CELEX data.^9^ In our trigram model, the expected length of words is represented by the probability of a special symbol marking the word-final position.^10^ Note that vowels can have negative representativeness for both Sublex≈_Germanic_ and Sublex≈_Latinate_ (e.g., ᾶː) when they have positive representativeness with respect to Sublex≈_-ity_.^11^ The comprehensive list of all uni- to trigram substrings is also available as Supplementary Material.^12^ The only possible counterexample to this generalization is *cheap*, which was built based on the Latin word *caupo* ‘small trader, innkeeper’. This Latin word, however, was adopted in early Proto-Germanic (Hoad, 2003), and thus is considered more adapted to the native phonotactics.^13^ We used the R function multinomial.test in the EMT package and executed the exact test.^14^ Although the CELEX database treats this suffix, *-s*, as an adjective-to-noun derivational morpheme, it might be more appropriate to analyze it as the inflectional plural marker.^15^ ✓DOC verbs are listed in § 2.1, Ex. 115 of Levin (1993); and *DOC-Lat verbs are found in §2.1, Ex. 118a.^16^ Levin’s (1993) list of *DOC-Lat verbs includes *broadcast*, but its components, *broad* and *cast*, are in fact both of Germanic origin (Stevenson & Lindberg, 2010). Accordingly, we excluded it from our main analysis. As a side note, this example was also classified into Sublex≈_Germanic_, so it did not contribute to adjudication between our model and the true etymology-based account (as both failed to predict the ungrammaticality of DOC).^17^ The etymological origin of the ✓DOC verbs were identified by referring to Wikipedia articles (see Appendix C for details) and the New Oxford American Dictionary (Stevenson & Lindberg, 2010). Consequently, we excluded etymologically ambiguous words, listed as “Latinates of Germanic origin” in Wikipedia, from the analysis. We also excluded *netmail* and *telex* because they are compounds/blends of Germanic and Latinate morphemes.^18^ Unlike *n*-gram and other autoregressive models (e.g., recurrent neural networks and Transformers), Optimality-Theoretic models typically define a probability distribution with a limited number of parameters (i.e., constraints). Consequently, they may fail to capture some of the stochastic differences among word clusters discovered by our model.^19^ Conversely, some researchers have argued that phonological features themselves emerge from statistical learning rather than being inherent to individual phonemes. Nelson (2022), for example, proposed an algorithm that groups phonemes based on their cooccurrence statistics, whose results aligned with traditional phonological features. This approach resembles the *n*-gram model, though its learned representations do not necessarily correspond to interpretable phonological features.^20^ Bayesian posterior distributions can also be estimated using Markov Chain Monte Carlo (MCMC) methods (Gilks et al., 1996; Hastings, 1970; Neal, 2000). However, these sampling-based approaches proved impractical for our clustering problem under realistic time constraints.^21^ https://en.wikipedia.org/wiki/List_of_English_words_of_Anglo-Saxon_origin, accessed on 5 April, 2019.^22^ https://en.wikipedia.org/wiki/List_of_English_words_of_Old_Norse_origin, accessed on 5 April, 2019.^23^ https://en.wikipedia.org/wiki/List_of_English_words_of_Dutch_origin, accessed on 5 April, 2019.^24^ https://en.wikipedia.org/wiki/List_of_Latin_words_with_English_derivatives, accessed on 5 April, 2019.^25^ https://en.wikipedia.org/wiki/List_of_English_words_of_French_origin_(A-C), accessed on 5 April, 2019.^26^ https://en.wikipedia.org/wiki/List_of_English_words_of_French_origin_(D-I), accessed on 5 April, 2019.^27^ https://en.wikipedia.org/wiki/List_of_English_words_of_French_origin_(J-R), accessed on 5 April, 2019.^28^ https://en.wikipedia.org/wiki/List_of_English_words_of_French_origin_(S-Z), accessed on 5 April, 2019.^29^ https://en.wikipedia.org/wiki/List_of_English_Latinates_of_Germanic_origin, accessed on 5 April, 2019.

## Supplementary Material



## Figures and Tables

**Figure D1.**
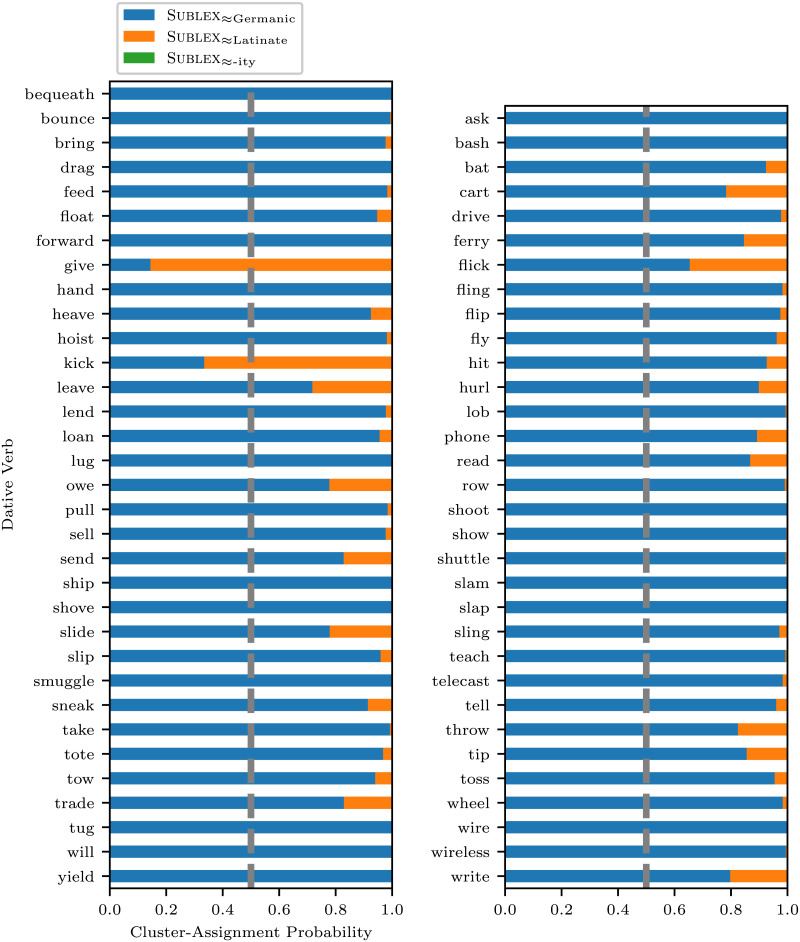
Cluster-assignment probabilities of ✓DOC-Lat dative verbs.

**Figure D2.**
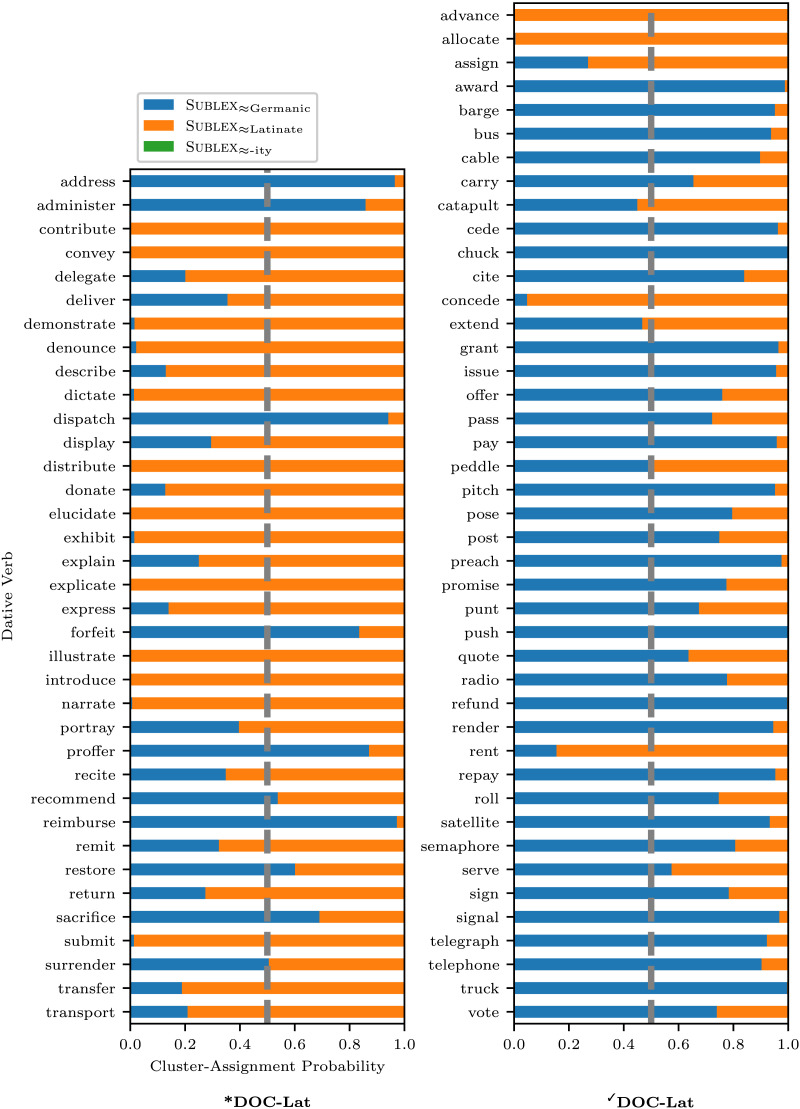
Cluster-assignment probabilities of *DOC-Lat (left) and ✓DOC-Lat (right) verbs.

## APPENDIX A: HYPERPARAMETERS

Our learning simulation of English word classes adopted exactly the same model that Morita and O’Donnell (2022) used for that of Japanese word clusters: the trigram model with backoff smoothing based on the hierarchical DP (HDP; Futrell et al., 2017; Goldwater et al., 2006; Teh et al., 2006), including the values on hyperparameters. Specifically, the concatenation parameters of the cluster assignment DP and the backoff HDP followed the gamma prior distribution Gamma(10, 10^−1^) (parameterized by the shape and scale), which is the standard setting also adopted by Futrell et al. (2017), Goldwater et al. (2006), Teh et al. (2006), etc. The top level unigram prior on the segments was uniform.

The parameters of the variational approximation of the posterior inference were also set in the same way as in (Morita & O’Donnell, 2022). Specifically, the upper bound on the number of word clusters was set to six, and that on the number of segment-generator distributions per backoff layer was set to twice the number of possible symbols: 52 phonetic segments plus two special symbols indicating the word-initial and -final positions. We optimized the variational approximator distributions using the coordinate ascent algorithm (Bishop, 2006; Blei & Jordan, 2006), and the best approximation result among 1000 runs with random initialization was reported here. Each run was terminated either when the improvement in the approximation error (measured by the evidence lower bound, or ELBO) per iteration became less than 0.1, or when the maximum number of iterations (= 5000) was reached.

## APPENDIX B: SUPPLEMENTARY INFORMATION ABOUT THE DATA FORMAT

As noted in the Data section, we trained our clustering model on the CELEX dataset (Baayen et al., 1995); Specifically, the training data was extracted from the epl.cd file of the dataset, while filtering out lemmas whose corpus frequency—reported in the “Cob” column—was zero. The phonetic transcription in this dataset is coded in a special format called *DISC*. DISC represents each distinct segment with a single ASCII letter, and our *n*-gram model treated each of them as a unit symbol. Specifically, we adopted the the “PhonStrsDISC” column of the epl.cd file, while the phonetic transcriptions in this paper have all been translated into IPA for better readability.

## APPENDIX C: IDENTIFICATION OF THE ETYMOLOGICAL ORIGIN

The CELEX database does not provide information on the etymological origin of words. Accordingly, to evaluate the alignment of our discovered clusters with the ground-truth etymology, we made use of a subset of words whose origin was identifiable in Wikipedia. Specifically, we considered words of Anglo-Saxon,^21^ Old Norse,^22^ Dutch,^23^ Latin,^24^ and French origin.^25^^,^^26^^,^^27^^,^^28^ Words with multiple origins were included in the data only if the origins were either all Germanic (Anglo-Saxon, Old Norse, or Dutch) or Latinate (Latin or French); words reported as “Latinates of Germanic origin”^29^ were excluded from the analysis. We also excluded words that were of ambiguous origin. The resulting data amounted to 14,172 words (consisting of 3,535 Germanics and 10,637 Latinates).

## APPENDIX D: PREDICTIONS OF DOC-GRAMMATICALITY FOR INDIVIDUAL VERBS

In the section titled “Syntactic Predictions from the Clustering Results”, we reported our model predictions regarding the DOC grammaticality of dative verbs in the form of word counts per grammaticality/etymology type × model-discovered cluster. Here, we provide more detailed results, reporting the cluster-assignment probability of each individual verb.

Figure D1 presents the posterior cluster-assignment probability of the non-Latinate dative verbs that allow DOC (✓DOC-Lat). The vertical dashed lines indicate 0.5, which serves as an approximate decision boundary ignoring the tiny probability mass on Sublex≈-ity.

We can see that the vast majority of them have a greater assignment probability to Sublex≈_Germanic_ (represented by the blue portions in the figure).

Latinate dative verbs prohibiting (*DOC-Lat) and permitting (✓DOC-Lat) DOC are listed in Figure D2 with their cluster-assignment probabilities. Most of the *DOC-Lat verbs had a greater probability of assignment to Sublex≈_Latinate_; by contrast, most of the ✓DOC-Lat verbs were MAP-classified into Sublex≈_Germanic_. And it is these “correct misclassifications of the exceptions” that made our model be a better predictor of DOC grammaticality than the true etymology.
